# Supplementary material for: Characteristics of Physicochemical Properties of Chalky Grains of Japonica Rice Generated by High Temperature during Ripening
Source: Foods. 2021 Dec 30;11(1):97. doi: 10.3390/foods11010097 (PMC8750872; doi:10.3390/foods11010097)
Supplement: Supplementary file 1 [file foods-11-00097-s001.zip › foods-1452357-supplementary.pdf]

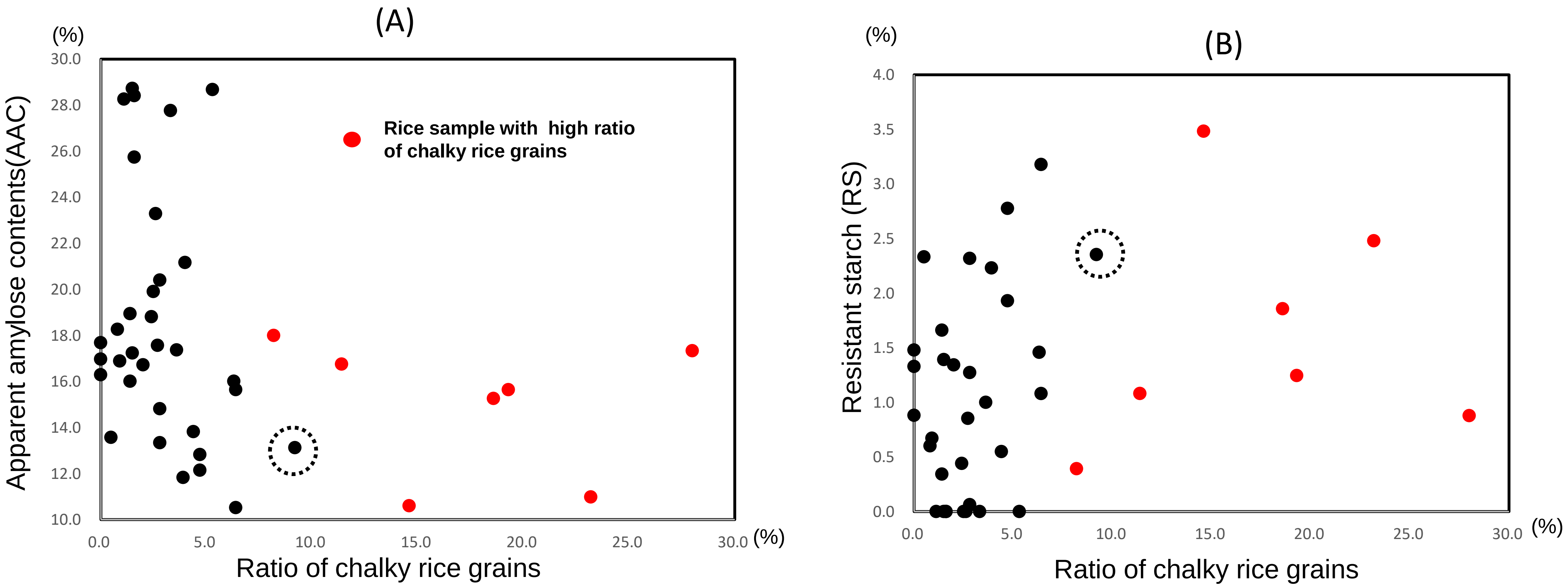


We selected 7 rice samples with a high ratio of chalky rice grains from 40 *Japonica* rice samples in 2019.

Dotted circle shows a high ratio of whole rice grains, so we did not select one.

Supplemental Figure S1. Relationship between ratios of chalky rice grains and apparent amylose contents (AACs), and resistant starch (RS)

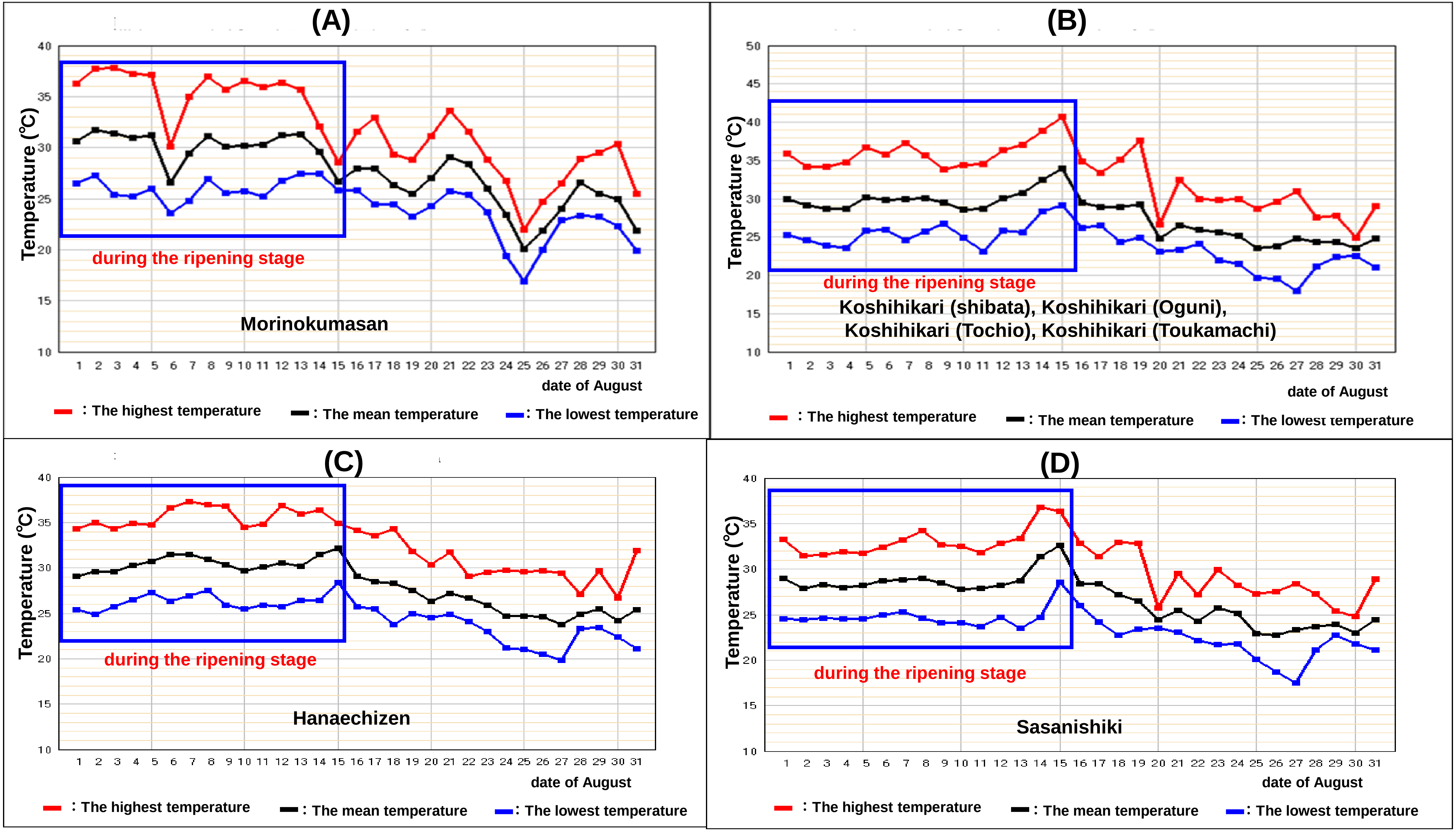


Supplemental Figure S2. The ambient temperature during the ripening stage of rice (August, 2019, Japan).
